# Dural lymphatics regulate clearance of extracellular tau from the CNS

**DOI:** 10.1186/s13024-019-0312-x

**Published:** 2019-02-27

**Authors:** Tirth K. Patel, LeMoyne Habimana-Griffin, Xuefeng Gao, Baogang Xu, Samuel Achilefu, Kari Alitalo, Celia A. McKee, Patrick W. Sheehan, Erik S. Musiek, Chengjie Xiong, Dean Coble, David M. Holtzman

**Affiliations:** 1 Department of Neurology, Hope Center for Neurological Disorders, Knight Alzheimer’s Disease Research Center, Washington University in St. Louis, St. Louis, MO 63110 USA; 2 Department of Radiology, Washington University, St. Louis, MO 63110 USA; 3 Department of Biochemistry and Molecular Biophysics, Department of Biomedical Engineering, Washington University, St. Louis, MO 63110 USA; 4 Wihuri Research Institute and Translational Cancer Biology Program, Biomedicum Helsinki, University of Helsinki, Helsinki, Finland; 5 Division of Biostatistics, Knight Alzheimer’s Disease Research Center, Washington University, St. Louis, MO 63110 USA

**Keywords:** Alzheimer’s disease, Tau, Tauopathy, Neurodegeneration, Dural lymphatic system, Glymphatic system, Tau clearance, Tau imaging

## Abstract

**Background:**

Alzheimer’s disease is characterized by two main neuropathological hallmarks: extracellular plaques of amyloid-β (Aβ) protein and intracellular aggregates of tau protein. Although tau is normally a soluble monomer that bind microtubules, in disease it forms insoluble, hyperphosphorylated aggregates in the cell body. Aside from its role in AD, tau is also involved in several other neurodegenerative disorders collectively called tauopathies, such as progressive supranuclear palsy (PSP), corticobasal degeneration (CBD), some forms of frontotemporal dementia, and argyrophilic grain disease (AGD). The prion hypothesis suggests that after an initial trigger event, misfolded forms of tau are released into the extracellular space, where they spread through different brain regions, enter cells, and seeding previously normal forms. Thus understanding mechanisms regulating the clearance of extracellular tau from the CNS is important. The discovery of a true lymphatic system in the dura and its potential role in mediating Aβ pathology prompted us to investigate its role in regulating extracellular tau clearance.

**Methods:**

To study clearance of extracellular tau from the brain, we conjugated monomeric human tau with a near-infrared dye cypate, and injected this labeled tau in the parenchyma of both wild-type and K14-VEGFR3-Ig transgenic mice, which lack a functional CNS lymphatic system. Following injection we performed longitudinal imaging using fluorescence molecular tomography (FMT) and quantified fluorescence to calculate clearance of tau from the brain. To complement this, we also measured tau clearance to the periphery by measuring plasma tau in both groups of mice.

**Results:**

Our results show that a significantly higher amount of tau is retained in the brains of K14-VEGFR3-Ig vs. wild type mice at 48 and 72 h post-injection and its subsequent clearance to the periphery is delayed. We found that clearance of reference tracer human serum albumin (HSA) was also significantly delayed in the K14-VEGFR3-Ig mice.

**Conclusions:**

The dural lymphatic system appears to play an important role in clearance of extracellular tau, since tau clearance is impaired in the absence of functional lymphatics. Based on our baseline characterization of extracellular tau clearance, future studies are warranted to look at the interaction between tau pathology and efficiency of lymphatic function.

## Background

Alzheimer's disease (AD) is the leading cause of dementia in the elderly and currently affects more than five million people in the United States. The two main neuropathological hallmarks of AD are extracellular plaques of amyloid-β (Aβ) and intracellular accumulations of aggregated, hyperphosphorylated forms of tau in structures such as neurofibrillary tangles (NFT) [1]. The amyloid cascade hypothesis holds that the triggering event in AD pathogenesis is the initial accumulation and aggregation of Aβ into oligomers and insoluble extracellular plaques [2]. This initiates a cascade that incites the misfolding and aggregation of soluble tau into insoluble forms, eventually leading to neurodegeneration. Loss of cognitive function in AD and other tauopathies is correlated with the amount of aggregated tau accumulation. In addition to its key role in AD pathology, tau has also been implicated in a host of other neurodegenerative disorders such as progressive supranuclear palsy (PSP), corticobasal degeneration (CBD), certain forms of frontotemporal dementia (FTD) and argyrophilic grain disease (AGD). Collectively termed tauopathies, these disorders all feature aggregated forms of tau in the CNS [3, 4].

One model explaining part of the pathogenesis of tauopathies is the prion hypothesis, which states that misfolded forms of tau can exit the cell, spread to distant regions of the brain where they can re-enter cells and “seed” previously normal forms of the protein, much like prion protein [5, 6]. Expression of mutant human tau in neurons in the entorhinal cortex shows spread of tau pathology to synaptically connected regions in the dentate gyrus of the hippocampus in mice [7, 8]. In addition to seeding and uptake in cell culture [9–12], injection of brain lysates from transgenic mice [13, 14], cell lysates [15], synthetic recombinant tau fibrils [16, 17] and tau extracts purified from human brains [18–22] into mouse models have also been shown to robustly induce uptake, seeding and spreading of tau pathology. If this model of tau propagation is correct, it is likely that extracellular tau plays a key role in mediating pathogenesis and progression of tauopathy. As a result understanding the mechanisms that regulate tau fate in the extracellular space of the CNS and its eventual clearance to the periphery is important.

Regulated clearance of substances out of the CNS to the periphery is vital to healthy functioning of the CNS, and as such is an important and active area of research. For all the attention focused on the brain in health and disease, little was known about fundamental clearance mechanisms until relatively recently. For decades, it was thought the brain enjoyed a ‘true’ immune privileged status because of an apparent lack of lymphatic drainage from the CNS. Early tracer injection studies showed that peptides and solutes injected in the CSF and parenchyma eventually make their way to deep cervical lymph nodes (dCLNs) by traveling along olfactory sinuses and the cribriform plates [23, 24].

More recent work, also largely done with tracers, refined this model further by proposing that solutes are cleared across paravascular routes and are aided in this process by astrocytes. The role of astrocytes in mediating this clearance led to this model being called the ‘glymphatic’ (a portmanteau of glia and lymphatic) system [25]. The water channel aquaporin 4 (AQP4), predominantly localized to astrocytic end-feet, was shown to be key to glymphatic clearance, as its deletion impaired clearance of solutes to dCLNs [26–28]. This model proposes that the interstitial fluid (ISF) compartment of the brain exchanges metabolites and macromolecules with the CSF compartment. This is driven by arterial pulsation [27], which causes solutes to exit the brain by following paravascular pathways aided by AQP4.

The characterization of this system has implications for aging and disease. Prolonged exposure to Aβ aggregates has been shown to impair glymphatic function in mouse models [29]. Deleting AQP4 leads to reduced transport of biomarkers of neuronal injury in a mouse model of TBI [28] and exacerbates existing tau pathology, presumably because tau aggregates are not cleared out of the brain properly [30].

More recent work has upended accepted dogma by conclusively showing that the brain does indeed have a ‘true’ lymphatic system responsible for draining macromolecules and cells from the deep parenchyma. These studies demonstrated and rigorously characterized a network of lymphatic vessels localized to the dura of the meninges using state of the art microscopy and high resolution fluorescent imaging [31, 32]. These dural lymphatics were shown to track along superior sagittal and transverse sinuses, ultimately draining into the deep cervical lymph nodes. Ablating dural lymphatics with either genetic manipulation or surgery resulted in significantly slower clearance of injected macromolecules in the deep parenchyma, perhaps indicating that the glymphatic and lymphatic system exist in parallel and might even be linked [32, 33]. Furthermore, it was shown that impaired lymphatic drainage can exacerbate amyloid pathology, particularly in the meninges [34].

Although it is known that extracellular clearance of Aβ is regulated by the transporters at the BBB such as RAGE, LRP1, LDLR and P-GP [35–39], by cellular enzymes such as neprilysin [40] and insulin-degrading enzyme [41], and by glymphatic flow [42, 43], data surrounding what regulates extracellular clearance of tau is limited. Extracellular tau in various CNS compartments can be detected in the periphery [30, 44, 45], but it is unclear what regulates this process. A recent study showed that various isoforms of tau are readily detected in plasma following injection in the ventricles [46] but the mechanisms mediating this process are unknown. There is also some evidence that the glymphatic system is involved [30]; however, the role of the dural lymphatic system has not yet been studied. This lack of information and proper understanding of tau clearance prompted us to investigate the role of dural lymphatic system in clearance of extracellular tau.

## Methods

### Animal surgeries and husbandry

Initial breeding pairs of K14-VEGFR3-Ig mice [47] were obtained from Dr. Kari Alitalo at the University of Helsinki and the colony was further expanded and maintained at Washington University School of Medicine. The transgenic mice are crossed with C57/BL6 mice. WT and TG mice used in all the experiments were littermates.

WT and K14-VEGFR3-Ig mice were anesthetized with ketamine/xylazine cocktail before being placed in the stereotactic surgery frame. Intrahippocampal injection of recombinant monomeric human tau (labeled and un-labeled) and human serum albumin (HSA) was performed at the following site: bregma − 2.5 mm, lateral 2 mm from midline, 2 mm ventral to the dura. All procedures were performed following protocols approved by the Animal Studies Committee and Washington University School of Medicine.

### Tau and HSA conjugation with cypate

Recombinant monomeric human tau (1N4R, tau-412, rPeptide) and HSA were conjugated with near-infrared dye cypate in the following manner: Cypate conjugation to proteins was carried out by NHS ester chemistry [48]. Cypate-NHS was prepared by dissolving 31.3 mg (0.05 mmol) cypate in 200 μl DMSO. Next a solution of 5.8 mg (0.05 mmol) NHS, and 4.8 mg (0.025 mmol) EDC was prepared in another 200 μl DMSO solution. Finally the two solutions were mixed and incubated overnight at room temperature. For conjugation, 100 μg tau was reconstituted with 90 μl pH 7.4 1 X PBS buffer. Then 0.75 μl Cypate-NHS was diluted with DMSO into 10 μl, and then added to the 90 μl tau solution and allowed to incubate for 2 h at room temperature.

For HSA conjugation 1.0 mg HSA was dissolved in 200 μl pH 7.4 1 X PBS buffer. Next 1.5 μl, Cypate-NHS DMSO solution was diluted with DMSO into 20 μl, added to the 200 μl HSA PBS solution, and then incubated at room temperature for overnight. Reaction mixtures were dialyzed with 1 X PBS, pH 7.4 at 4 °C for overnight, and then lyophilized. Dye and protein concentrations were determined by protein assay (Bio-Rad) and UV-Vis absorption spectrum, to give dye to protein molar ratio.

### In vivo FMT of tau-cypate and HSA-cypate drainage

In vivo FMT was performed on a Perkin Elmer FMT4000 imaging system. Following intra-hippocampal injection of cypate labeled tau, mice were anesthetized with 2% isoflurane for imaging. Both WT and K14-VEGFR3-Ig mice were imaged at 1, 2, 24, 48, 72 and 168 h post-injection of tau. A 2 μl of solution (1.1 μg) of monomeric human tau or HSA was injected at a flow-rate of 0.2 μl/min. Fluorescence was quantified based on instrument calibration with cypate phantoms. Total fluorescence of tau and HSA fluorescent conjugates was measured in manually drawn ROIs approximating the brain and lymph nodes. To control for variability caused by injection, data for each mouse at each time point were normalized to the fluorescent signal at the 1 h time point.

### Plasma tau measurement

For plasma measurement, 50 mg/kg of anti-human antibody HJ 8.5 was injected intraperitoneally as described in [44]. Mice were injected with 2 μl of solution (1.1 μg) of unlabeled, monomeric tau 1 h post-antibody administration. Blood was collected at 2, 24, 48, 72, 168 h post-tau injection. Samples were spun at 8000 g for 10 min to obtain plasma. The Simoa HD-1 analyzer (Quanterix Corp) was used to measure human tau in plasma as previously described [44].

### Meningeal extraction and immunohistochemistry

Mice were anesthetized by intraperitoneal injection of pentobarbital (200 mg/kg) for perfusion with ice-cold Dulbecco’s PBS with Heparin. Following perfusion, the skull cap was carefully removed and fixed overnight in 4% paraformaldehyde. Intact meninges were then peeled off and stored as floating sections in PBS until immunohistochemistry. For LYVE1 staining, meninges were washed in PBS-Triton X-100 (0.5%) and blocked in PBS-T with 0.5% BSA at room temperature. Sections were incubated with LYVE1-e660 (ThermoFisher) at 1:200 overnight at 4^0^ C. Sections were washed in PBS-T prior to mounting on slides in Prolong Gold antifade reagent with DAPI (ThermoFisher) mounting medium. Slides were imaged on Zeiss Axio Imager Z2 fluorescence microscope and Cytation 5 imaging reader (Biotek Imaging, Inc.). Images were processed on ZEN software suite (Carl Zeiss, Inc.) and Cytation 5 imaging reader.

### Statistics

Bulk of data plotting and statistical analysis were done on GraphPad Prism 5. For tau-cypate (*n* = 6–9) and HSA-cypate (*n* = 4–6) injection and brain retention studies, a two-way ANOVA with a Bonferroni post-test by was used to analyze the two groups of mice at each time point (i.e. 2, 24, 48, 72 and 168 h). For plasma tau measurement a mixed effects linear model was used to compare plasma tau (pg/ml) for the two groups (WT *n* = 5, K14-VEGFR3-Ig *n* = 6) across five times (2, 24, 48, 72, and 168 h). Akaike’s AIC was used to evaluate 15 ovariance structures to determine the best fit model for the two analyses. Least square estimates of the differences between groups (i.e., mouse types or drug treatment) at each time period were used to compare the trajectory of response over time. All tests were conducted at the alpha = 0.05 level of significance. All tests were run in PROC MIXED of SAS 9.4.

## Results

### Extracellular tau clearance is impaired and significantly more tau is retained in the brain of K14-VEGFR3-Ig mice

**Fig. 1:**
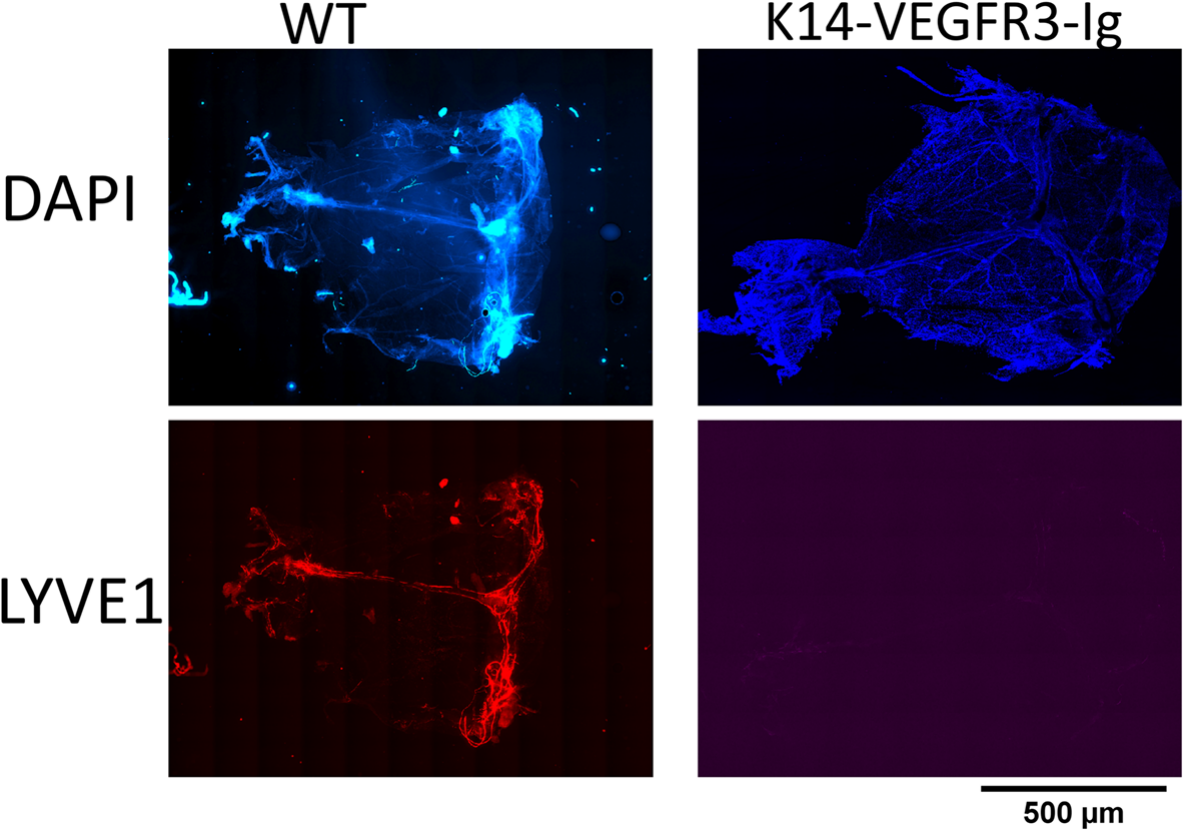
Histological characterization of the dural lymphatic system. Intact meninges from both WT and K14-VEGFR3-Ig mice were stained for cellular marker DAPI (top) and the lymphatic endothelial marker LYVE1 (bottom). Representative sections are shown here. WT mice show the characteristic distribution of dural lymph vessels along the superior sagittal and transverse sinuses (as delineated by LYVE1 staining). K14-VEGFR3-Ig mice have no LYVE1 staining

We utilized the K14-VEGFR3-Ig transgenic mice for our studies. These mice lack a functional dural lymphatic system [32] and thus provide a model to study the contribution of this system to clearance of substances from the brain. We first confirmed that these mice lack dural lymphatics by immunohistochemistry for LYVE1, a lymph endothelial cell marker (Fig. 1). WT mice show clear distribution of lymphatic vessels along superior sagittal and transverse sinuses, while K14-VEGFR3-Ig mice have no LYVE1 staining, indicating a complete lack of lymphatic vessels.

**Fig. 2:**
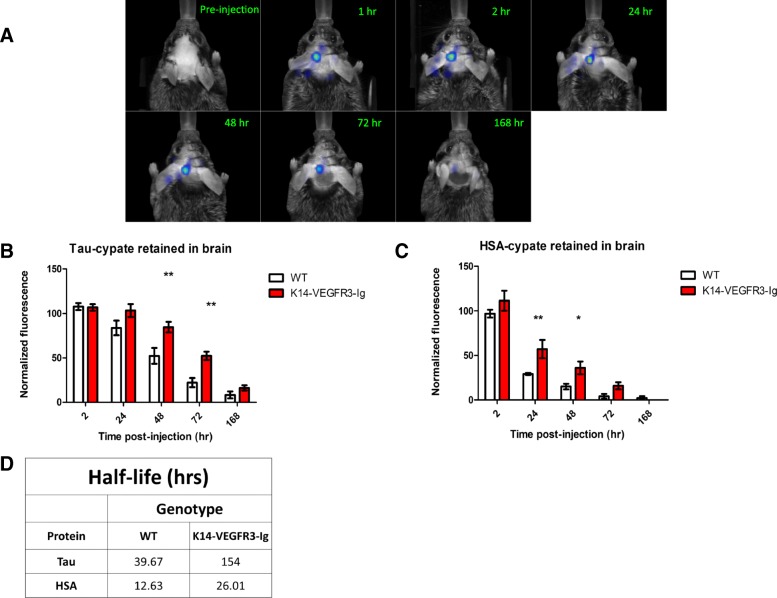
K14-VEGFR3-Ig mice retain significantly more tau in the brain following intra-CNS injection. **a** Panel showing distribution and gradual clearance of tau-cypate (as measured by longitudinal FMT) in a representative WT mouse from pre-injection through 168 h post-injection. Monomeric recombinant human tau was conjugated with the near-infrared fluorescent dye cypate and stereotactically injected in the hippocampus of WT and K14-VEGFR3-Ig mice. Mice were longitudinally imaged with FMT at the time-points indicated. The same injection and imaging timeline was followed for experiments involving clearance of HSA-cypate in WT and K14-VEGFR3-Ig mice **b** Significantly more tau is retained in the brain of K14-VEGFR3-Ig mice, indicating delayed clearance. 4–6 mo old WT (*n* = 9; 5 males, 4 females) and K14-VEGFR3-Ig mice (*n* = 6; 3 males, 3 females) were injected with tau-cypate in the hippocampus. They were imaged with FMT at 1, 2, 24, 48, 72 and 168 h post injection. The amount of fluorescence in the brain was quantified and normalized to values for the 1 h time point. A significantly higher amount of tau was retained in the K14-VEGFR3-Ig mice compared to WT mice at 48 h (85% vs 52% retention) and 72 h (52% vs 22% retention), following injection. Data was analyzed by a two-way ANOVA with a Bonferroni post-test. (** *p*-value < 0.01). **c** Significantly more HSA is retained in the brain of K14-VEGFR3-Ig mice, indicating delayed clearance. 4–6 mo old WT (*n* = 4; 1 male, 3 females) and K14-VEGFR3-Ig mice (*n* = 6; 4 males, 2 females) were injected with HSA-cypate in the hippocampus. They were imaged with FMT at 1, 2, 24, 48, 72 and 168 h post injection. The amount of fluorescence in the brain was quantified and normalized to values for the 1 h time point. A significantly higher amount of HSA was retained in the K14-VEGFR3-Ig mice compared to WT mice at 24 h (57% vs 29% retention) and 48 h (36% vs 15% retention) following injection. Data was analyzed by a two-way ANOVA with a Bonferroni post-test. (** *p*-value < 0.01, * *p*-value < 0.05). **d** Table summarizing half-life of tau and HSA in the CNS following their injection in both WT and K14-VEGFR3-Ig mice. The half-life of both proteins is longer in the K14-VEGFR3-Ig mice

To assess clearance of extracellular tau from the CNS over time, we performed serial FMT – an optical imaging modality that allows for in vivo imaging of fluorescent probes through layers of skin, bone and tissue – of mice injected with recombinant monomeric human tau labeled with cypate, a near-infrared dye that can be easily conjugated to a number of different tracers, probes and molecules for optical imaging of tumors and other physiological processes [49–52]. In this experiment, 4-6mo old WT and K14-VEGFR3-Ig mice were imaged at 1, 2, 24, 48, 72 and 168 h post-injection of tau (Fig. 2a). Fluorescence was quantified in the brain to calculate amount of tau retained at each time point. Signal for each mouse at each time point was normalized to the 1 h time point. As we imaged mice over several days, we observed a clear result: tau clearance is delayed from the brain in K14-VEGFR3-Ig mice. Significantly more tau is retained at 48 h (85% vs 52%) and 72 h (52% vs 22%) post-injection in the K14-VEGFR3-Ig mice, strongly suggesting that in the absence of dural lymphatics, extracellular tau is not as effectively cleared from the CNS to the periphery (Fig. 2b). This is reflected in the half-life of tau in both groups (Fig. 2d): tau has a shorter half-life of 39.67 h in WT mice compared to 154 h in the K14-VEGFR3-Ig mice.

### Clearance of HSA from CNS is also impaired in K14-VEGFR3-Ig mice

To get a more clear understanding of tau clearance by lymphatics we repeated our experiment by injecting HSA-cypate to serve as a reference tracer. Since it is known that albumin is cleared by lymphatic vessels out of the CNS [31, 32], we hypothesized delayed clearance of HSA in K14-VEGFR3-Ig mice. Longitudinal FMT of 4–6 mo WT and K14-VEGFR3-Ig mice injected with HSA-cypate reveals that significantly more HSA is retained in the brain of the transgenic mice at 24 h (57% vs 29%) and 48 h (36% vs 15%) compared to WT mice (Fig. 2c), suggesting that similar to tau, clearance of HSA is delayed in mice lacking a functional dural lymphatic system. Additionally we found that half-life of HSA was longer in K14-VEGFR3-Ig mice (26 h) compared to WT mice (~ 13 h). This is in agreement with published reports of rat albumin’s half-life in rat brain [53, 54] being in the range of 11–18 h, thus further validating this method to measuring CNS clearance of proteins. The fact that HSA is cleared faster than tau could perhaps indicate uptake and/or additional extracellular retention of tau within CNS.

### Measurement of plasma tau reveals a trend toward delayed clearance in K14-VEGFR3-Ig mice

**Fig. 3:**
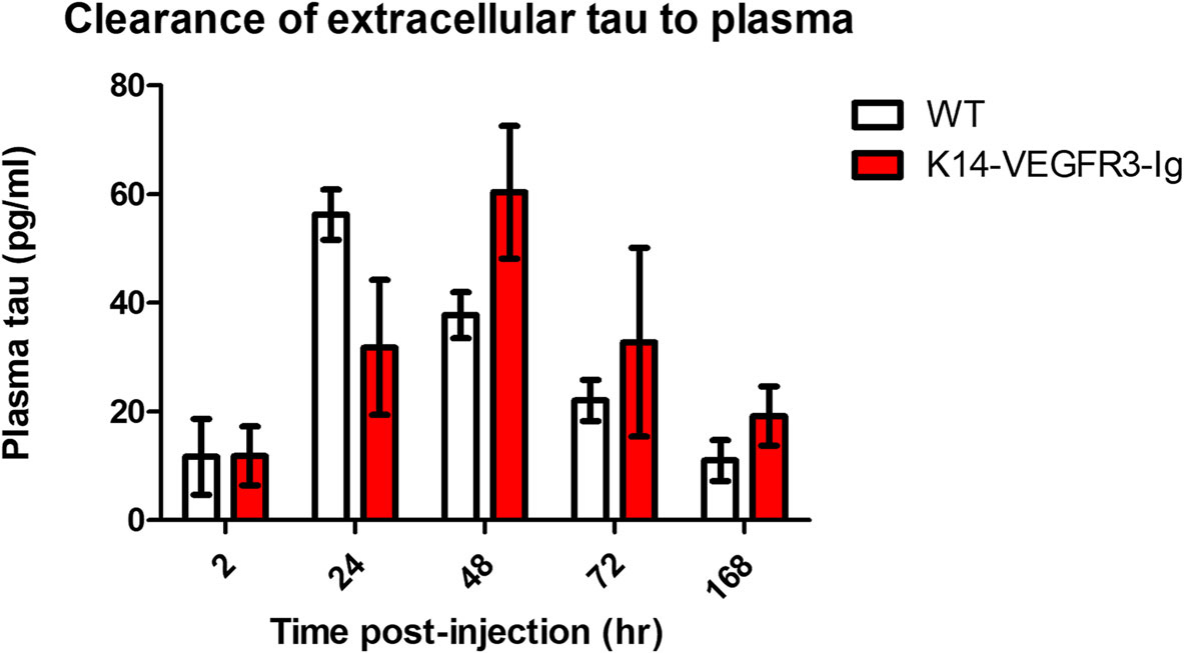
K14-VEGFR3-Ig mice show delayed clearance of extracellular tau to the plasma after intra CNS injection. 4-6mo old WT (*n* = 6; 3 males, 3 females) and K14-VEGFR3-Ig mice (*n* = 5; 3 males, 2 females) were injected with anti-tau antibody HJ 8.5 to stabilize tau entering the plasma from the CNS to allow for its measurement. An hour later recombinant monomeric human tau was injected in the hippocampus and blood was collected at time points indicated. Plasma tau was measured using the ultrasensitive Simoa HD1-Analyzer platform. Though the overall difference between tau for the two mouse groups was not significant (*p* = 0.6766), the interaction between the two mouse groups over time was significant (*p* = 0.0191). Plasma tau peaks earlier in WT mice compared to K14-VEGFR3-Ig mice (24 vs 48 h). Amount of plasma tau is significantly higher in K14-VEGFR3-Ig mice at 48 h compared to WT mice (*p* = 0.0260), indicative of delayed clearance of tau due to impaired lymphatics. Data was analyzed by mixed effects linear model. Akaike’s AIC was used to evaluate 15 covariance structures to determine the best fit model for this analysis. Least square estimates of the differences between the two mouse groups at each time period were used to compare the trajectory of response over time

As we showed there is delayed clearance of extracellular tau from the brain, one would predict that the amount of tau appearing in the blood would also be delayed. In order to study this directly, we measured plasma tau in both groups of mice to complement FMT experiments looking at brain retention of tau to get a more complete picture of tau clearance. We used an antibody-mediated approach [44] to increase half-life of soluble tau in the plasma to allow us to measure it reliably. This was done because the half-life of tau once it reaches the plasma is only 8.49 min; however, in the presence of the anti-tau antibody HJ8.5, the half-life of plasma tau increases to 3.4 h allowing one to measure it there in a more facile fashion [44]. Following intraperitoneal antibody administration of HJ 8.5 (an anti-human tau antibody), WT and K14-VEGFR3-Ig mice were injected in the hippocampus with 1.1 μg tau. Blood was collected at 2, 24, 48, 72 and 168 h post-injection and plasma human tau was measured by a high-sensitivity ELISA on a Simoa analyzer. The plasma tau assay uses capture antibody HJ8.7 (which binds to N-terminal epitopes 117–122 of tau) and biotinylated detection antibody BT2 (which binds to epitopes 194–198 of tau). We found that K14-VEGFR3-Ig mice, which retained a significantly higher amount of tau in the brain, correspondingly also show delayed appearance of tau in the periphery (Fig. 3) compared to WT mice. The amount of human tau in the plasma peaked earlier in the WT mice than in the K14-VEGFR3-Ig mice (24 h vs. 48 h). Significantly higher plasma tau was present in K14-VEGFR3-Ig mice at 48 h, when the WT mice had already cleared most of the tau from the periphery. Together with the fluorescence data from the brain, these plasma tau measurements strongly suggest that impaired dural lymphatic function leads to increased retention in the brain as well as delayed clearance of tau to the periphery.

## Discussion

While intracellular forms of tau are critical for its normal function as well as its role in neurodegenerative diseases, extracellular forms of tau appear to have an important role in the process of tau aggregate spreading transynaptically in different tauopathies as well as possibly in a component of tau toxicity. Given this, it is important to understand what regulates the clearance of extracellular tau from the brain. One important recently identified pathway that is involved in extracellular protein clearance from the brain is the dural lymphatics. Because of the recent discovery of bona fide lymph vessels in the meninges, we particularly focused on their role in this process. By using the K14-VEGFR3-Ig mouse model, which lacks dural lymphatics and shows delayed clearance of CNS macromolecules and tracers [32], we demonstrated that extracellular tau clearance from the CNS is significantly impaired in the absence of a functioning dural lymphatic system. A significantly higher amount of tau is retained in the brain of the K14-VEGFR3-Ig mice and its eventual clearance and appearance in the periphery is also delayed.

As our work was done to establish ‘baseline’ clearance mechanism of monomeric tau, it opens several exciting future directions: does disruption of lymphatic vessels exacerbate tau pathology? What effect does age have on lymphatic clearance of tau? Is clearance of aggregated tau markedly different than monomeric tau? The mice we used in our work are otherwise healthy and do not show any neuropathological alterations (such as tau aggregation). As a result it would be interesting to determine whether the clearance of tau is altered in the presence of significant AD or other pathology as this will help us to understand if there is a link between CNS lymphatics and neurodegeneration.

Since the glymphatic system appears to play a key role in the ISF-CSF exchange of solutes, we can propose a model of extracellular tau clearance that traces the fate of ISF tau: following its release into the ISF compartment, tau is cleared into the CSF space by the glymphatic system. Once in the CSF it is drained to the cervical lymph nodes by the dural lymphatic system. Thus the glymphatic and lymphatic systems most likely work in tandem to accomplish the eventual clearance of tau to the periphery.

However one key observation from our FMT experiments in K14-VEGFR3-Ig mice is that despite complete lack of lymphatic vessels, extracellular tau is still able to be cleared out of the brain to the periphery, albeit at a much slower rate than in healthy WT mice. This implies the existence of alternate paths for tau clearance and/or uptake within the CNS. Coupled with the fact that passive clearance of HSA appears to be faster than passive clearance of tau (despite their similar molecular weights) this leads to the hypothesis that the dural lymphatic system might show some specificity in transporting macromolecules out of the CNS. The potential existence of alternate clearance mechanisms needs to be investigated further. K14-VEGFR3-Ig mice lack a functional lymphatic system owing to lack of signaling through the VEGFR3 receptor. Mice show complete lack of lymph vasculature and profound lymphedema. They survive the neonatal period despite these defects and subsequently even show regeneration of lymph vasculature in the periphery. In adulthood they only lack the dural lymphatic system [32, 47]. Though these mice do not have any defects in vascular permeability that are detectable, it is possible that abnormal development of peripheral lymphatics might also lead to disturbance in degradation and eventual clearance of plasma tau.

Finally, in the process of investigating the contribution of dural lymphatics to extracellular clearance we made extensive use of FMT, a technique that has previously been used mostly to study tumor evolution and other related cellular processes in vivo [49]. We have thus established a novel use for this versatile technique, which can hopefully be used for more extensive clearance studies in the future.

## Conclusions

We demonstrate a novel use of FMT in studying clearance of extracellular tau from the CNS. Using a mouse model lacking functional lymphatics we performed longitudinal FMT to show that tau clearance is notably impaired in the absence of functional lymphatics and its eventual clearance to the periphery is significantly delayed, thus establishing a key role for the dural lymphatic system in mediating extracellular tau clearance. Our study highlights a likely link between the glymphatic and lymphatic systems in mediating extracellular clearance and opens new avenues to investigate the interplay between neurodegeneration and the dural lymphatic system.

## Abbreviations

AD: Alzheimer's disease
AGD: Argyrophillic grain disease
AQP4: Aquaporin 4
Aβ: Amyloid beta
BBB: Blood brain barrier
CBD: Corticobasal degeneration
CNS: Central nervous system
CSF: Cerebrospinal fluid
dCLN: Deep cervical lymph node
FMT: Fluorescence molecular tomography
FTD: Frontotemporal dementia
HSA: Human serum albumin
ISF: Interstitial fluid
LDLR: Low density lipoprotein recetor
LRP1: Low density lipoprotein receptor-related protein 1
NFT: Neurofibrillary tangle
P-GP: P-glycoprotein
PSP: Progressive supranuclear palsy
RAGE: Receptor for advanced glycation end products
WT: Wild-type

## References

[1] Holtzman DM, Mandelkow E, Selkoe DJ (2012). Alzheimer Disease in 2020. Cold Spring Harb Perspect Med.

[2] Musiek ES, Holtzman DM (2015). Three dimensions of the amyloid hypothesis: time, space and “wingmen”. Nat Neurosci.

[3] Mandelkow EM, Mandelkow E (2012). Biochemistry and cell biology of tau protein in neurofibrillary degeneration. Cold Spring Harb Perspect Biol.

[4] Li C, Götz J (2017). Tau-based therapies in neurodegeneration: opportunities and challenges. Nat Rev Drug Discov.

[5] Jucker M, Walker LC (2013). Self-propagation of pathogenic protein aggregates in neurodegenerative diseases. Nature..

[6] Davis AA, Leyns CEG, Holtzman DM (2018). Intercellular spread of protein aggregates in neurodegenerative disease. Annu Rev Cell Dev Biol.

[7] de Calignon A, Polydoro M, Suárez-Calvet M, William C, Adamowicz DH, Kopeikina KJ (2012). Propagation of tau pathology in a model of early Alzheimer’s disease. Neuron..

[8] Liu L, Drouet V, Wu JW, Witter MP, Small SA, Clelland C (2012). Trans-synaptic spread of tau pathology in vivo. PLoS One.

[9] Holmes BB, Furman JL, Mahan TE, Yamasaki TR, Mirbaha H, Eades WC (2014). Proteopathic tau seeding predicts tauopathy in vivo. Proc Natl Acad Sci.

[10] Kfoury N, Holmes BB, Jiang H, Holtzman DM, Diamond MI (2012). Trans-cellular propagation of tau aggregation by fibrillar species. J Biol Chem.

[11] Frost B, Jacks RL, Diamond MI (2009). Propagation of tau misfolding from the outside to the inside of a cell. J Biol Chem.

[12] Wu JW, Herman M, Liu L, Simoes S, Acker CM, Figueroa H (2013). Small misfolded tau species are internalized via bulk endocytosis and Anterogradely and Retrogradely transported in neurons. J Biol Chem.

[13] Ahmed Z, Cooper J, Murray TK, Garn K, McNaughton E, Clarke H (2014). A novel in vivo model of tau propagation with rapid and progressive neurofibrillary tangle pathology: the pattern of spread is determined by connectivity, not proximity. Acta Neuropathol.

[14] Clavaguera F, Hench J, Lavenir I, Schweighauser G, Frank S, Goedert M (2014). Peripheral administration of tau aggregates triggers intracerebral tauopathy in transgenic mice. Acta Neuropathol.

[15] Sanders DW, Kaufman SK, DeVos SL, Sharma AM, Mirbaha H, Li A (2014). Distinct tau prion strains propagate in cells and mice and define different Tauopathies. Neuron..

[16] Iba M, Guo JL, McBride JD, Zhang B, Trojanowski JQ, Lee VM-Y (2013). Synthetic tau fibrils mediate transmission of neurofibrillary tangles in a transgenic mouse model of Alzheimer’s-like tauopathy. J Neurosci.

[17] Peeraer E, Bottelbergs A, Van Kolen K, Stancu I-C, Vasconcelos B, Mahieu M (2015). Intracerebral injection of preformed synthetic tau fibrils initiates widespread tauopathy and neuronal loss in the brains of tau transgenic mice. Neurobiol Dis.

[18] Clavaguera F, Akatsu H, Fraser G, Crowther RA, Frank S, Hench J (2013). Brain homogenates from human tauopathies induce tau inclusions in mouse brain. Proc Natl Acad Sci U S A.

[19] Boluda S, Iba M, Zhang B, Raible KM, Lee VM-Y, Trojanowski JQ (2014). Differential induction and spread of tau pathology in young PS19 tau transgenic mice following intracerebral injections of pathological tau from Alzheimer’s disease or corticobasal degeneration brains. Acta Neuropathol.

[20] He Z, Guo JL, McBride JD, Narasimhan S, Kim H, Changolkar L (2018). Amyloid-β plaques enhance Alzheimer’s brain tau-seeded pathologies by facilitating neuritic plaque tau aggregation. Nat Med.

[21] Lasagna-Reeves CA, Castillo-Carranza DL, Sengupta U, Guerrero-Munoz MJ, Kiritoshi T, Neugebauer V (2012). Alzheimer brain-derived tau oligomers propagate pathology from endogenous tau. Sci Rep.

[22] Guo JL, Narasimhan S, Changolkar L, He Z, Stieber A, Zhang B (2016). Unique pathological tau conformers from Alzheimer’s brains transmit tau pathology in nontransgenic mice. J Exp Med.

[23] Cserr HF, Harling-Berg CJ, Knopf PM (1992). Drainage of brain extracellular fluid into blood and deep cervical lymph and its immunological significance. Brain Pathol.

[24] Rennels ML, Gregory TF, Blaumanis OR, Fujimoto K, Grady PA (1985). Evidence for a ‘Paravascular’ fluid circulation in the mammalian central nervous system, provided by the rapid distribution of tracer protein throughout the brain from the subarachnoid space. Brain Res.

[25] Xie L, Kang H, Xu Q, Chen MJ, Liao Y, Thiyagarajan M (2013). Sleep drives metabolite clearance from the adult brain. Science..

[26] Iliff JJ, Lee H, Yu M, Feng T, Logan J, Nedergaard M (2013). Brain-wide pathway for waste clearance captured by contrast-enhanced MRI. J Clin Invest.

[27] Iliff JJ, Wang M, Zeppenfeld DM, Venkataraman A, Plog BA, Liao Y (2013). Cerebral arterial pulsation drives Paravascular CSF-interstitial fluid exchange in the murine brain. J Neurosci.

[28] Plog BA, Dashnaw ML, Hitomi E, Peng W, Liao Y, Lou N (2015). Biomarkers of traumatic injury are transported from brain to blood via the Glymphatic system. J Neurosci.

[29] Peng W, Achariyar TM, Li B, Liao Y, Mestre H, Hitomi E (2016). Suppression of glymphatic fluid transport in a mouse model of Alzheimer’s disease. Neurobiol Dis.

[30] Iliff JJ, Chen MJ, Plog BA, Zeppenfeld DM, Soltero M, Yang L (2014). Impairment of Glymphatic pathway function promotes tau pathology after traumatic brain injury. J Neurosci.

[31] Louveau A, Smirnov I, Keyes TJ, Eccles JD, Rouhani SJ, Peske JD (2015). Structural and functional features of central nervous system lymphatic vessels. Nature..

[32] Aspelund A, Antila S, Proulx ST, Karlsen TV, Karaman S, Detmar M (2015). A dural lymphatic vascular system that drains brain interstitial fluid and macromolecules. J Exp Med.

[33] Louveau A, Plog BA, Antila S, Alitalo K, Nedergaard M, Kipnis J (2017). Understanding the functions and relationships of the glymphatic system and meningeal lymphatics. J Clin Invest.

[34] Da Mesquita S, Louveau A, Vaccari A, Smirnov I, Cornelison RC, Kingsmore KM (2018). Functional aspects of meningeal lymphatics in ageing and Alzheimer’s disease. Nature..

[35] Deane R, Du Yan S, Submamaryan RK, LaRue B, Jovanovic S, Hogg E (2003). RAGE mediates amyloid-β peptide transport across the blood-brain barrier and accumulation in brain. Nat Med.

[36] Castellano JM, Deane R, Gottesdiener AJ, Verghese PB, Stewart FR, West T (2012). Low-density lipoprotein receptor overexpression enhances the rate of brain-to-blood a clearance in a mouse model of -amyloidosis. Proc Natl Acad Sci.

[37] Shibata M, Yamada S, Kumar SR, Calero M, Bading J, Frangione B (2000). Clearance of Alzheimer’s amyloid-β1-40 peptide from brain by LDL receptor–related protein-1 at the blood-brain barrier. J Clin Invest.

[38] Elali A, Rivest S (2013). The role of ABCB1 and ABCA1 in beta-amyloid clearance at the neurovascular unit in Alzheimer’s disease. Front Physiol.

[39] Tarasoff-Conway JM, Carare RO, Osorio RS, Glodzik L, Butler T, Fieremans E (2015). Clearance systems in the brain—implications for Alzheimer disease. Nat Rev Neurol.

[40] Iwata N, Tsubuki S, Takaki Y, Shirotani K, Lu B, Gerard NP (2001). Metabolic regulation of brain Abeta by neprilysin. Science..

[41] Qiu WQ, Walsh DM, Ye Z, Vekrellis K, Zhang J, Podlisny MB (1998). Insulin-degrading enzyme regulates extracellular levels of amyloid beta-protein by degradation. J Biol Chem.

[42] Iliff JJ, Wang M, Liao Y, Plogg BA, Peng W, Gundersen GA (2012). A Paravascular pathway facilitates CSF flow through the brain parenchyma and the clearance of interstitial solutes, including amyloid. Sci Transl Med.

[43] Kress BT, Iliff JJ, Xia M, Wang M, Wei HS, Zeppenfeld D (2014). Impairment of paravascular clearance pathways in the aging brain. Ann Neurol.

[44] Yanamandra K, Patel TK, Jiang H, Schindler S, Ulrich JD, Boxer AL (2017). Anti-tau antibody administration increases plasma tau in transgenic mice and patients with tauopathy. Sci Transl Med.

[45] Shahim P, Tegner Y, Wilson DH, Randall J, Skillbäck T, Pazooki D (2014). Blood biomarkers for brain injury in concussed professional ice hockey players. JAMA Neurol.

[46] Banks WA, Kovac A, Majerova P, Bullock KM, Shi M, Zhang J (2016). Tau proteins cross the blood-brain barrier. J Alzheimers Dis.

[47] Mäkinen T, Jussila L, Veikkola T, Karpanen T, Kettunen MI, Pulkkanen KJ (2001). Inhibition of lymphangiogenesis with resulting lymphedema in transgenic mice expressing soluble VEGF receptor-3. Nat Med.

[48] Nanda JS, Lorsch JR (2014). Labeling of a protein with fluorophores using maleimide derivitization. Methods Enzymol.

[49] Zhang X, Bloch S, Akers W, Achilefu S. Near-Infrared Molecular Probes for In Vivo Imaging. Curr Protoc Cytom. 2012;Chapter 12:Unit12.27. 10.1002/0471142956.cy1227s60 PMC3334312 22470154

[50] Achilefu S, Dorshow RB, Bugaj JE, Rajagopalan R (2000). Novel receptor-targeted fluorescent contrast agents for in vivo tumor imaging. Investig Radiol.

[51] Ye Y, Li WP, Anderson CJ, Kao J, Nikiforovich GV, Achilefu S (2003). Synthesis and characterization of a macrocyclic near-infrared optical scaffold. J Am Chem Soc.

[52] Ye Y, Bloch S, Kao J, Achilefu S (2005). Multivalent carbocyanine molecular probes: synthesis and applications. Bioconjug Chem.

[53] Cserr HF, Cooper DN, Suri PK, Patlak CS (1981). Efflux of radiolabeled polyethylene glycols and albumin from rat brain. Am J Physiol Physiol.

[54] Yamada S, DePasquale M, Patlak CS, Cserr HF (1991). Albumin outflow into deep cervical lymph from different regions of rabbit brain. Am J Physiol Circ Physiol.

